# Formulation, Physicochemical Optimization, and Forensic Evaluation of Zinc Oxide- and Curcumin-Loaded Solid Lipid Nanoparticles for Safe Fingerprint Detection in Forensic Medicine

**DOI:** 10.3390/ph19060904

**Published:** 2026-06-06

**Authors:** Ahmed A. Katamesh, Rehab Abdelmonem, Sarah A. Khater, Hadel A. Abo El-Enin, Abdullah A. Alshehri, Noran Khaled, Khadiga A. Fattah, Inas Essam Ibrahim Al-Samadi

**Affiliations:** 1 Department of Pharmaceutics, College of Pharmacy, University of Ha’il, Ha’il 81442, Saudi Arabia; a.katamsh@uoh.edu.sa; 2 Department of Industrial Pharmacy, College of Pharmaceutical Sciences and Drug Manufacturing, Misr University for Science and Technology (MUST), Giza 12585, Egypt; inas.samadi@must.edu.eg; 3 Department of Forensic Medicine and Clinical Toxicology, College of Medicine, Misr University for Science and Technology (MUST), 6th of October City 12566, Egypt; sarah.khater@must.edu.eg (S.A.K.); khadiga.abdelfattah@must.edu.eg (K.A.F.); 4 Department of Pharmaceutics, Egyptian Drug Authority (EDA), Giza 12511, Egypt; 5 Department of Clinical Pharmacy, College of Pharmacy, Taif University, Taif 21944, Saudi Arabia; a.aalshehri@tu.edu.sa; 6 Demonstrator of Forensic Medicine and Clinical Toxicology, Misr University for Science and Technology (MUST), Giza 12585, Egypt; nouranfarouke95@hotmail.com

**Keywords:** ZnO, curcumin, SLNs, fingerprints, nano-forensics

## Abstract

**Purpose**: Nano-forensics is the latest application of nano-based technology for the purpose of fingerprint detection to improve precision, expedite investigations, and enhance safety. Solid lipid nanoparticles (SLNs) represent a promising pharmaceutical nanocarrier system for different applications. This study focused on applying ZnO and/or curcumin nanoparticles (NPs) to SLNs for the purpose of fingerprint detection to improve their sensitivity, safety and selectivity. **Methods**: A factorial design was utilized to select the optimized Cur-SLNs and ZnO-SLNs on the basis of the smallest particle size (PS), the lowest polydispersity index (PDI) and the highest zeta potential (ZP) value. To select the safe SLN-NPs, a cytotoxicity test was applied and they were compared to the most commonly applied product in fingerprint detection. The optimized formula was investigated according to the morphological structure; confocal spectroscopy and a stability study at different storage conditions were applied. Then the SLN-NPs were evaluated for their sensitivity, efficacy and selectivity in fingerprint detection. **Results**: The obtained optimal Cur-SLNs and ZnO-SLNs showed a nano PS of 221.55 ± 1.34 nm and 313.950 ± 1.87 nm, respectively, a PDI value < 0.7 and a ZP > 20 mV. The cytotoxicity data demonstrate that Cur-SLNs have low toxicity, so they will be the chosen formula. TEM and Raman spectroscopy analysis of the optimized Cur-SLN formulation validated the encapsulation efficiency and structural integrity of the pharmaceutical nanosystem. Furthermore, the powder showed stability and good results with higher adherence but smudged the prints on surfaces due to the slightest moisture. **Conclusions**: Overall, the results confirmed that Cur-SLN nanopowders can be developed as a suggested alternative to the current toxic powders used for latent fingerprint detection in forensic science, but only after further research on various surfaces and in different conditions.

## 1. Introduction

For over a century, fingerprints have served as a primary method of personal identification in forensic science [1]. During the twentieth century, their use became widespread, particularly in criminal investigations and record-keeping systems. At crime scenes, offenders often leave behind various types of physical evidence, among which fingerprints are some of the most accessible and reliable. Traditional powdering techniques have long been used to collect these impressions effectively. In recent decades, visualization techniques have advanced, including the use of chemical reagents and high-intensity light sources [2].

There has been growing recognition of the detailed features present in the ridge patterns of the palmar surfaces of the hands. Beyond basic characteristics like ridge endings and bifurcations, additional features such as sweat pores, ridge edge shapes, scars, and creases are now considered valuable. The analysis and use of these finer details for identification purposes is referred to as Ridgeology [3]. When the friction ridges of the skin come into contact with a surface, they leave behind impressions known as latent fingerprints. These prints may become invisible over time and therefore require appropriate detection techniques [3]. In conventional powdering methods, fine particles adhere selectively to the residues left by fingerprints, creating contrast between the ridges and the background surface [4]. Zinc oxide nanopowders, particularly in dried form, have shown notable effectiveness in forensic applications. Studies indicate that these nanoparticles are efficient for developing latent fingerprints on non-porous materials, often producing fluorescent images when exposed to ultraviolet light [5].

Innovative materials with improved qualities have been developed as a result of nanotechnology’s capacity to modify materials at the atomic and molecular scale. Nanotechnology has found use in forensic science, or nano-forensics, due to its wide range of applications [6]. The application of nanoscale technology for research is the main emphasis of this new field [7]. Particles with distinct physical and chemical properties ranging in size from 1 to 100 nanometers are the focus of nanotechnology [8].

Fingerprints remain one of the most common and critical forms of evidence found at crime scenes, as they directly link individuals to criminal activity. Recent developments involving nanoparticles (NPs) have significantly advanced fingerprint detection methods, leading to what is now referred to as nano-fingerprinting [9].

Modern research has explored techniques such as micro-X-ray fluorescence for visualizing latent fingerprints. Unlike traditional chemical approaches—including iodine fuming, ninhydrin, silver nitrate, and cyanoacrylate—this method offers distinct advantages. However, it still requires specialized equipment and trained personnel, which can limit its practical use [10].

Consequently, nano-based technologies offer promising alternatives for on-site fingerprint detection with reduced error rates. In addition, conventional fingerprint detection techniques, such as powdering, rely on chemical or physical contact with fingerprint remains [11,12]. Despite their widespread usage, these techniques have several health and environmental drawbacks, including a dependence on potentially dangerous chemicals, limited environmental adaptation, and low sensitivity to old or rough surfaces [13]. The majority of fingerprint development powders contain chemicals that are harmful to long-term health. Curcumin and semi-spherical zinc oxide were potentially used for developing latent fingerprints due to their accessibility. But their health concerns and low visibility, respectively, encourage the development of an alternative form.

A more sophisticated approach is provided by nanotechnology. Because of their tiny size and large surface area, nanoscale particles interact with fingerprint remnants more precisely. Stronger contrast, improved resolution, and greater versatility across different surfaces can be achieved by these nanomaterials by binding to certain residue components [14]. It offers suggestions that protect our environment and the health of forensic professionals in addition to improving the safety and effectiveness of nanoparticles in forensic applications [15]. For example, zinc oxide nanoparticles not only produce clear fingerprint images but also exhibit natural fluorescence under UV light, particularly in moist conditions. Enhanced fluorescence has also been observed under long-wave UV illumination [11,16].

Curcumin-based powders have also been investigated for latent fingerprint development and have shown effectiveness in personal identification, particularly in theft-related cases [17]. Curcumin is derived from turmeric (Curcuma longa), a perennial plant belonging to the Zingiberaceae family, widely cultivated in India. The rhizomes of this plant produce turmeric, which contains curcumin as its primary active compound, along with related derivatives in varying proportions [18].

The physical characteristics of nanoparticles, such as size and morphology, play a crucial role in determining their optical behavior and suitability for fingerprint detection [19]. This has encouraged the development of ZnO (zinc oxide) and curcumin (Cur) nanoparticles for forensic applications. Among various nanomaterials, solid lipid nanoparticles (SLNs) offer several advantages, including biocompatibility, biodegradability, low toxicity, and the ability to deliver both hydrophilic and lipophilic substances in a controlled manner [20,21]. Due to their stable structure and cost-effectiveness, SLNs are also considered promising materials for optical biosensing applications in forensic science [22].

An exhaustive search was done and no previous studies were found applying nano-technology based on ZnO-NPs or curcumin-NPs in SLNs for the purpose of fingerprint detection. Additionally, this study will be the first to test this technology in Egypt. Given that nanoparticle size and structure significantly influence their optical performance, this study aims to load ZnO and curcumin into SLN systems to improve fingerprint detection and analysis directly at crime scenes with minimal error. To the best of our knowledge, this research represents the first attempt to compare the effectiveness of SLNs in enhancing the sensitivity, selectivity, and safety of ZnO and curcumin nanoparticles for latent fingerprint visualization.

## 2. Results and Discussion

### 2.1. Preparation and Characterization of ZnO and Cur-Loaded SLNs

Obtaining nanoparticles with uniform nanosize and optimum stability is the primary objective of the ZnO and Cur-loaded SLN nanovesicle preparation process. The resulting nanovesicles are easier to produce and analyze fingerprint evidence immediately with the least amount of mistakes. The performance and interaction of nanoparticles with forensic analytes are significantly influenced by their size, shape, and surface charge, which can cause challenges. Because of their tiny size and large surface area, the nanostructures interact with fingerprint residues more precisely [23,24]. As a result, the primary focus of the investigation was the effectiveness of fingerprint detection. Because curcumin is readily available, inexpensive, and less harmful, it was selected as an example of organics powders, which are obtained from natural sources—food material—which have the potential to generate latent fingerprints. ZnO particles, on the other hand, were employed as metal-based organic powders that stick to latent fingerprints and improve their visibility on various surfaces [25]. Additionally, particle size, release behavior, stability during storage, and drug loading can all be impacted by the use of suitable surfactants [26]. The encapsulation state, which itself an outcome of the physicochemical optimization parameters (PS, PDI, ZP), is already included in the design as the response of the chosen factors. Optimizing for the smallest, most homogeneous, and most stable nanoparticles inherently favors the molecular dispersion state [27].

All factors had a substantial impact on the tested responses, namely, API type and quantity and SAA type. They are with a non-significant lack of fit, as shown in Table 1 and Table 2. Furthermore, the findings of the statistical analysis (ANOVA) show that, at the 95% level of significance, all linear factors corresponding to the variables under investigation have a significant impact on every tested response. At the same level, their interaction was also determined to be substantial. The potential of the model created to predict the experimental findings and the closeness of the anticipated and experimental results (difference < 0.2) are guaranteed by the high R^2^. An appropriate accuracy, or the models’ capacity to cover the design space, is indicated by a signal-to-noise ratio greater than 4 [28]. Table 3 displays the resultant equations in terms of coded factors.

**Figure 1:**
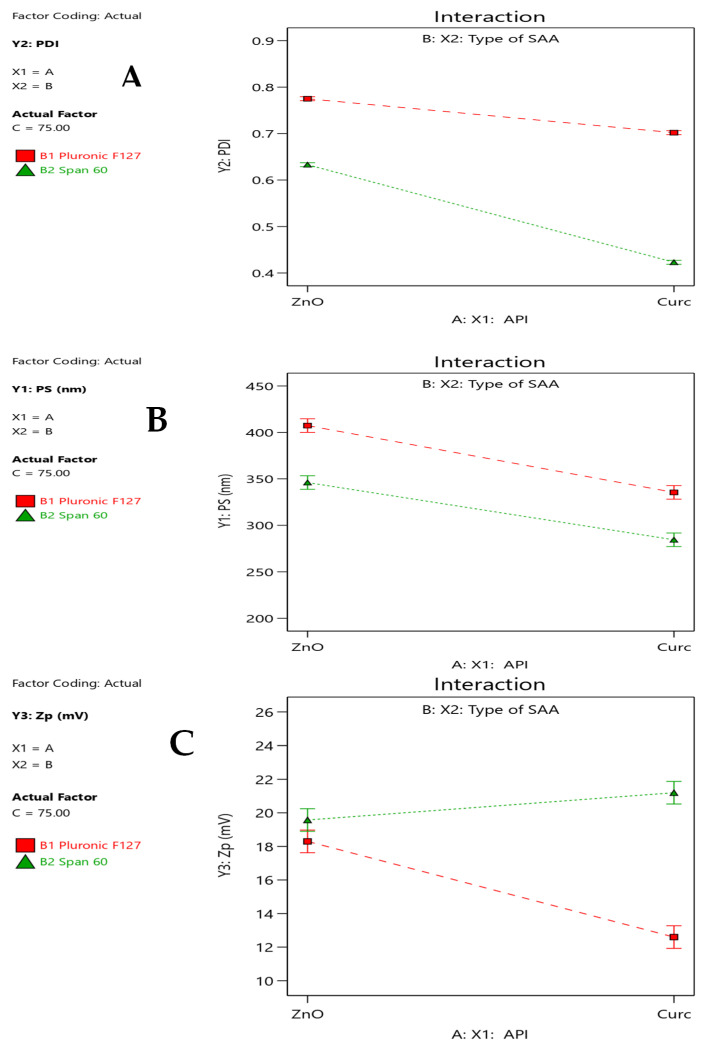
The main effects of formulation variables on (**A**): polydispersity index (PDI), (**B**): particle size (PS), and (**C**): zeta potential (ZP). Increasing drug amount concentration (mg/mL) had a positive influence on particle size and a negative effect on ZP value, while a negative effect was seen by changing the SAA type on the particle size and PDI, with a positive effect on ZP value when changing the API, which had a negative effect on all tested factors (PDI, PS and ZP value).

The PDI varied from 0.416 to 0.815, as seen in Table 1. By measuring the size of the generated nanoparticle system distribution, the polydispersity index (PDI) indicates the degree of homogeneity in any colloidal system [29]. A PDI value less than 0.7 indicates a more narrow particle size distribution and the formation of a more homogeneous, monodisperse system [30]. As represented in Figure 1A and Table 3, changing ZnO to Cur and SAA type has a negative effect on the PDI value, indicating the formation of more uniform particles. The opposite effect occurs, with the drug amount increasing, which increases the PDI values. The same result was observed for PS, where all of the formulations had a nanoparticle size ranging from 214.7 nm to 430.1 nm (Table 3 and Figure 1B).

The latter could be attributed to the fact that the different drugs have different pore-penetrating abilities. The high-molecular-weight drug was represented in curcumin, 368.38 g/mol, compared to ZnO, 81.38 g/mol, which led to a more homogeneous size distribution reflected as a decrease in the PDI [31]. Additionally, the type of surfactant in SLNs is an important factor for the determination of the physicochemical characteristics. A low PDI indicating partial separation of nanoparticles is observed, suggesting that the particle aggregation begins to reduce into individual particles and become more distinct and well-dispersed [32]. The results indicate an efficiency of Span 60 to produce more homogenous particles than PF127. Although the interaction between the API type and amount have a positive effect on increasing PDI value, it was to a very low extent (0.0025, Table 3), which could be negligible.

PS was measured by using the dynamic light scattering (DLS) method demonstrate that all formulation have a suitable PS < 500 nm. For PS distribution, changing the SAA from PF127 to Span 60 led to a decrease in PS. This was attributed to the higher viscosity of the outer phase introduced by PF127, which eventually reduced the diffusion rates of lipid molecules and led to the formation of bigger particles [33]. Adding more drugs, in agreement with previous reports, led to an increase in the amount of entrapped drugs, therefore increasing the PS [34]. Therefore, the interaction between different factors led to an increase in the PS.

The zeta potential value indicates the surface charge potential, which maintains the nanoparticles’ physical stability by reducing the chance of aggregation in the produced formulation. By increasing particle repulsion, van der Waals attractive forces can be resisted, avoiding particle aggregation [35].

The prepared SLNs have accepted positive Z-potential values that ranged from 12.3 ± 1.11 to 24.2 ± 1.09 mV (Table 1). All factors have a significant effect on the zeta potential value. As represented, the emulsifier type not only affects the particle size but also the formulation stability. The emulsifier type (hydrophilic and lipophilic surfactants) was used in the formulation to improve their stability [36]. As represented in Table 1 and Figure 1C, changing the SAA from a hydrophilic type (PF127) to a hydrophobic one (Span 60) significantly improves the system stability, which was correlated to the PDI results.

API amount and type produce a significant decrease in ZP value. There was a significant interaction between the used API and SAA, mainly curcumin and Span 60, which occurred in the SLN preparation, increasing the zeta potential value and thus the system stability. This can be attributed to the substantial lipophilicity of the cur and Span 60, which are incorporated, compared to ZnO and PF127 [37]. Although the observed ZP values are below the conventional |±30 mV| threshold for purely electrostatic stability, the low PDI values across all formulations suggest adequate dispersion stability, attributable to combined electrostatic and steric mechanisms [38].

### 2.2. Optimization of the ZnO and Cur-Loaded SLNs

**Figure 2:**
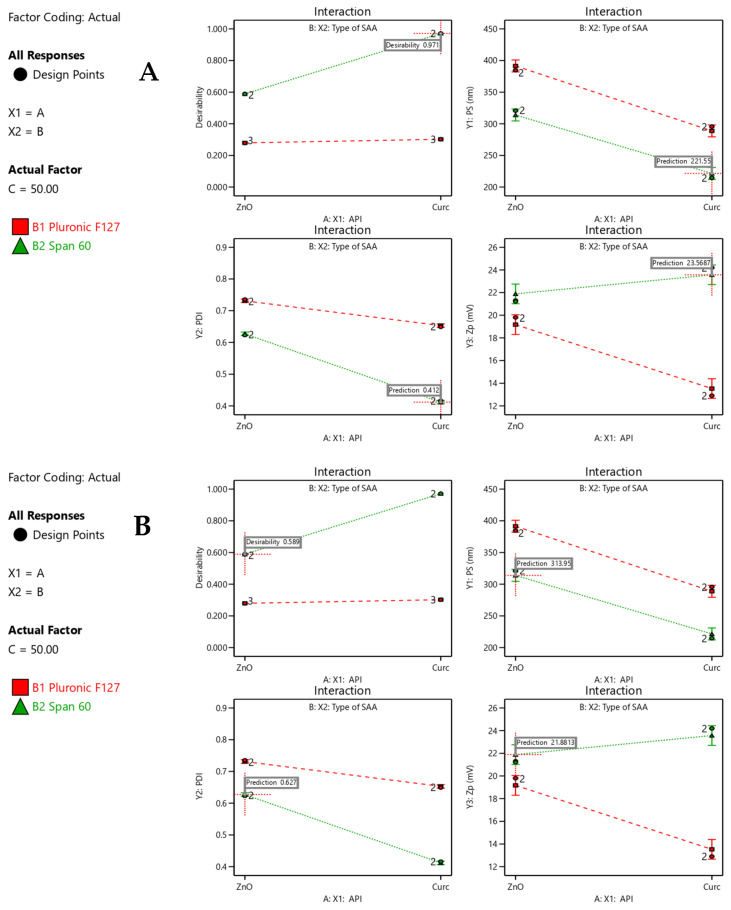
The interaction plot represents the values of coding factors when optimized to fulfill the target product profile criteria: minimum particle size, PDI and maximum ZP value. (**A**) For Cur-SLN selected formula. (**B**) For ZnO-SLN selected formula. PS and PDI were measured by DLS while ZP was measured by electrophoresis and all are expressed as mean ± SD (*n* = 3).

Parameter optimization was performed by applying response constraints to predict the most suitable variable values based on maximum desirability. The formulation obtained from this process was subsequently subjected to full characterization. The absence of any significant residual error confirms the reliability and validity of the numerical optimization approach employed in this study. The composition of the optimized system is presented in Figure 2.

Experimental findings revealed that the optimized curcumin-loaded formulation (50 mg/mL curcumin with Span 60) exhibited a computed high desirability value (0.971, Figure 2A). It produced nanoparticles with a mean diameter of 221.55 ± 1.34 nm and a polydispersity index (PDI) of 0.412. The zeta potential exceeded 20 mV (23.569 ± 1.38), reflecting enhanced electrostatic repulsion between particles, which minimizes aggregation and supports good colloidal stability [39].

The ZnO-SLNs prepared under identical conditions (50 mg/mL using Span 60, Figure 2B) exhibited a desirability value of 0.589 and has also demonstrated acceptable characteristics, including a particle size of 313.950 ± 1.87 nm, a PDI of 0.627 (within the acceptable limit of <0.7), and a zeta potential above 20 mV (21.88 ± 1.8). Because nanoparticles have an incredibly high surface area–mass ratio [25], they bind effectively to fingerprint residues, increasing contrast and clarity while the zeta value of NPs is a criterion influencing the interaction and maintains the system stability [4]. Through electrostatic attraction to negatively charged carboxylate groups inside sebaceous fingerprint residue components, the positive surface charge also provides a forensic adhesion advantage [40]. Accordingly, both formulations were considered appropriate candidates for subsequent investigations.

### 2.3. Cytotoxicity Evaluation

**Figure 3:**
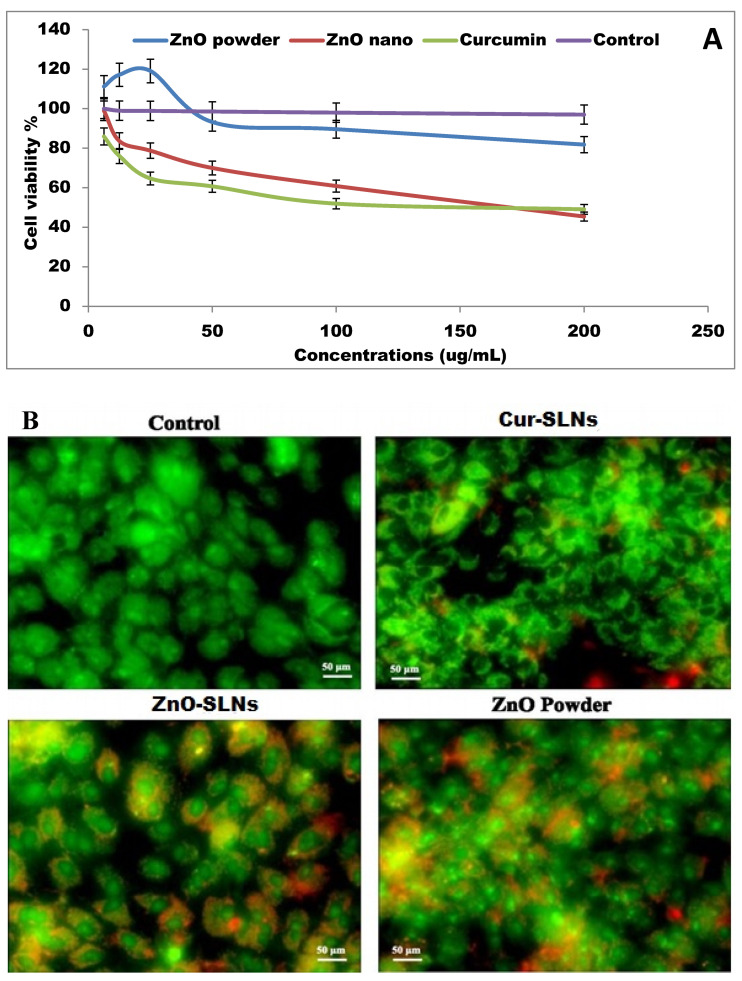
Cytotoxicity study. (**A**) Cell viability assay on hFB cells after incubation with Cur-SLNs, ZnO powder and ZnO-SLNs at increasing drug concentrations (6.25–200 μM). (**A**) Cell viability was assessed using the MTT assay, and results are presented as the % of viable cells compared to the untreated cells. Data points are expressed as mean ± SD (*n* = 5). (**B**) The fluorescence photos of hFB cells stained with Acridine Orange/Ethidium Bromide (AO/EtBr) stain after treatment with Cur-SLNs, ZnO nano- and ZnO powder, all at 150 µg/mL for 24 h in comparison to the control cells. The curcumin caused delayed apoptosis together with a few necrotic cells. The ZnO-SLNs and ZnO powder caused extensive early and late apoptosis with minimal necrotic changes. Both forms caused chromatin condensations in almost all nuclei. The scale bar is 50 µm.

Before any nanocarrier can be used in clinical settings, the possible carrier toxicity must be investigated. The safety of nanocarriers was the main concern for their applications [39,41]. Using hFB cells, cytotoxicity was tested at dosages ranging from 6.25 to 200 μM. Every compound studied had a dose-dependent impact, as seen in Figure 3. Based on Figure 3A, the IC50 values for Cur-SLNs, ZnO-SLNs (150 µg/mL), and commercial ZnO Powder (150 µg/mL) were determined to be 51.41 μg/mL, 53.24 μg/mL, and 86.53 μg/mL, respectively. The significant decrease in the IC50 for the prepared Cur-SLNs relative to the others is remarkable and indicative of the formulations’ ability to enhance the cellular uptake [41]. On the other hand, SLN-encapsulated materials required a lower concentration to inhibit 50% of cell growth in this in vitro model. This result should be interpreted carefully: curcumin is a well-characterized anti-proliferative agent whose activity in cell culture may contribute to the apparent cytotoxicity signal independent of any toxic mechanism relevant to forensic exposure scenarios. The acute forensic exposure route (topical surface application) and concentration are substantially different from the sustained cell culture conditions used here; therefore, direct extrapolation to practitioner safety requires caution.

Cell death profile where qualitatively assessed when compared to the control cells, the fluorescence images in Figure 3B of stained hFB cells treated with Cur-SLNs (150 µg/mL), ZnO-SLNs (150 µg/mL), and ZnO Powder (150 µg/mL) for 24 h show that the curcumin produced a delay in apoptosis, even if there were relatively predominantly early apoptotic morphology, with a few necrotic cells. ZnO powder and ZnO-SLNs, on the other hand, produced significantly more extensive early and late apoptosis with few necrotic alterations, and nearly all nuclei had chromatin condensations. Necrotic cell fractions (yellow fluorescence) were most prominent in the ZnO powder-treated group. This pattern suggests that Cur-SLNs are associated with a less necrotic, more regulated cell death mechanism than ZnO-based formulations at equivalent concentrations, which has implications for tissue irritation potential in practical forensic use.

### 2.4. Evaluation of Optimized Cur-SLNs

#### 2.4.1. Morphology Characterization of the Selected Cur-SLNs

**Figure 4:**
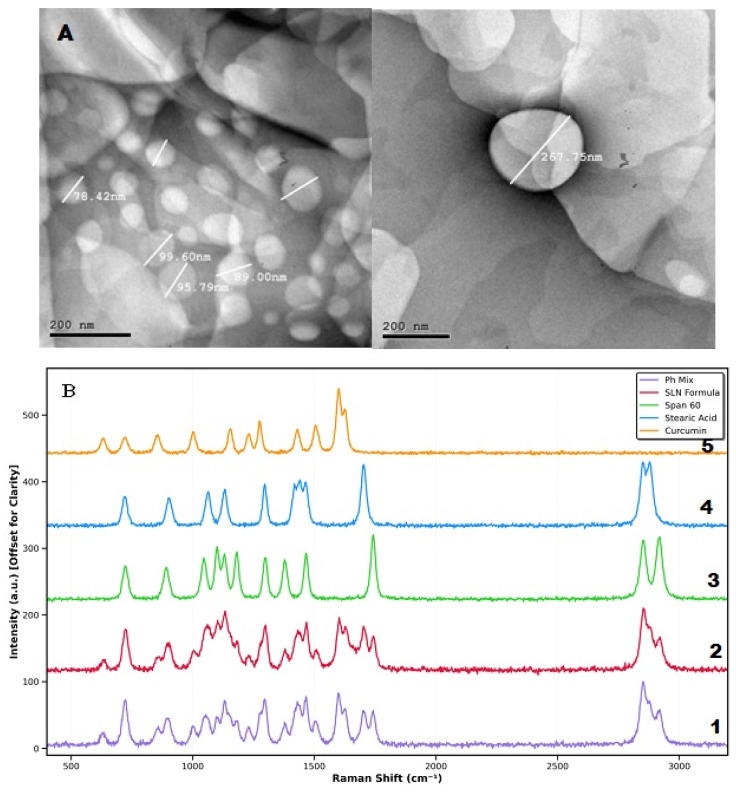
Evaluation of the optimized Cur-SLNs. (**A**): Morphological characterization of the optimized formula; Cur-SLNs by transmission electron micrography. Cur-SLNs appeared as spherical non-aggregate nanostructures. (**B**): Raman spectra of (1) crystalline Cur and (2) strearic acid, (3) Span 60, (4) Cur-SLNs and (5) a physical mixure of all components.

TEM images of the ideal Cur-SLNs showed smooth-surfaced, spherical, well-defined vesicles that did not aggregate (Figure 4A). Additionally, the TEM image’s measurement of the optimal Cur-SLN diameter was well matched with Malvern Zetasizer’s observations.

#### 2.4.2. Raman Spectroscopy Analysis of Optimized Cur-SLN Formulation

The comprehensive Raman spectroscopic investigation of curcumin-loaded solid lipid nanoparticles and their constituent components provides molecular-level insights into drug–lipid interactions, formulation structure, and nanoparticle characteristics. This non-destructive analytical approach successfully elucidated critical distinctions between physical admixture and formulated nanocarrier systems, validating encapsulation efficiency and structural integrity of the pharmaceutical nanosystem.

The Raman spectrum of pure curcumin exhibited characteristic vibrational signatures consistent with its polyphenolic aromatic structure (Figure 4B(1)). Prominent peaks were observed at 1628 cm^−1^ and 1603 cm^−1^, attributed to aromatic C=C stretching vibrations of the benzene rings conjugated with the heptadienone backbone [15,16]. Additional significant bands appeared at 1599 cm^−1^ (aromatic ring vibration), 1506 cm^−1^ (C=C stretching), and 1430 cm^−1^ (aromatic C-C stretching mode). The intense peak at 1276 cm^−1^ corresponded to aromatic C-O stretching vibration of methoxy substituents, while the band at 1154 cm^−1^ was assigned to C-O stretching coupled with aromatic ring deformation. Lower frequency regions displayed characteristic peaks at 1002 cm^−1^ (ring breathing mode), 855 cm^−1^ (aromatic C-H out-of-plane bending), and 720 cm^−1^ (aromatic ring deformation). These spectral features provided a diagnostic fingerprint for curcumin identification and subsequent monitoring of drug–lipid interactions [42].

Comparative Raman spectroscopic analysis between the SLN formulation and physical mixture reference revealed critical molecular-level distinctions indicative of drug–lipid interactions and nanostructure formation (Figure 4B(4,5)). Both samples displayed spectral contributions from all three components (curcumin, stearic acid, Span 60; Figure 4B(1–3)), yet notable differences in relative peak intensities, bandwidths, and subtle frequency shifts distinguished the formulated system from simple physical admixture.

The aromatic C=C stretching region of curcumin (1600–1650 cm^−1^) exhibited reduced relative intensity and slight broadening in the SLN formulation compared to the physical mixture, suggesting intimate molecular-level mixing and possible π-π stacking interactions within the lipid matrix [43,44]. The characteristic curcumin peak at 1628 cm^−1^ demonstrated a marginal frequency shift (<3 cm^−1^) in the SLNs system, indicating an altered vibrational environment due to encapsulation within the hydrophobic lipid core. Similarly, the aromatic C-O stretching band at 1276 cm^−1^ showed reduced sharpness in the formulation, consistent with disruption of crystalline curcumin structure upon dissolution in the lipid phase. The latter indicates transition from crystalline to amorphous or molecularly dispersed state within the lipid phase [45,46] and facilitates drug accommodation [47,48]. This transformation is thermodynamically favorable for enhancing drug solubility and preventing recrystallization during storage, thereby improving pharmaceutical performance. The balance between crystallinity (providing physical stability) and disorder (enabling drug-loading capacity) is a critical consideration in SLN formulation optimization.

In the aliphatic C-H stretching region (2800–3000 cm^−1^), the SLN formulation displayed subtly modified peak profiles compared to the physical mixture. The CH_2_ symmetric stretching band at approximately 2850 cm^−1^ showed slight broadening and asymmetry, reflecting conformational heterogeneity of lipid chains in the nanoparticulate state versus bulk crystalline lipid [49,50]. The intensity ratio between asymmetric (2917 cm^−1^) and symmetric (2850 cm^−1^) CH_2_ stretching modes differed between samples, providing indirect information regarding lipid chain organization and molecular packing density within nanoparticles.

The carbonyl stretching region revealed particularly informative spectral modifications. The SLN formulation exhibited a broad composite band centered around 1705–1720 cm^−1^, representing contributions from both stearic acid carboxylic acid C=O (1700 cm^−1^) and Span 60 ester C=O (1738 cm^−1^). The relative contributions of these components differed from those in the physical mixture, suggesting altered hydrogen bonding environments and molecular interactions within the nanostructure. Peak broadening in this region indicated increased structural disorder and molecular environment heterogeneity characteristic of amorphous or semi-crystalline lipid phases in nanoparticles [51]. Span 60 demonstrates dual functionality in SLN systems: reducing interfacial tension during nanoparticle formation and providing steric stabilization against aggregation during storage [52]. The broadening of this feature suggests increased heterogeneity in hydrogen bonding environments, consistent with surfactant molecules distributed on the nanoparticle surface, thereby creating interfacial regions with modified lipid organization [53].

### 2.5. Effect of Storage Conditions on Optimized CUR-SLNs

The results of the stored formulae did not differ significantly (paired *t*-test, *p* > 0.05) from those of the freshly prepared formula, indicating that the optimized Cur-SLNs were stable when stored at 4 °C and 25 °C over three months, as evidenced by statistical evaluation of PS, ZP, and the PDI (Table 4). This outcome was consistent with other studies showing the increased stability of lipid-based nanoparticles, particularly SLNs [54].

### 2.6. Fingerprint Detection Study of Curcumin SLNs in Forensic Medicine

**Figure 5:**
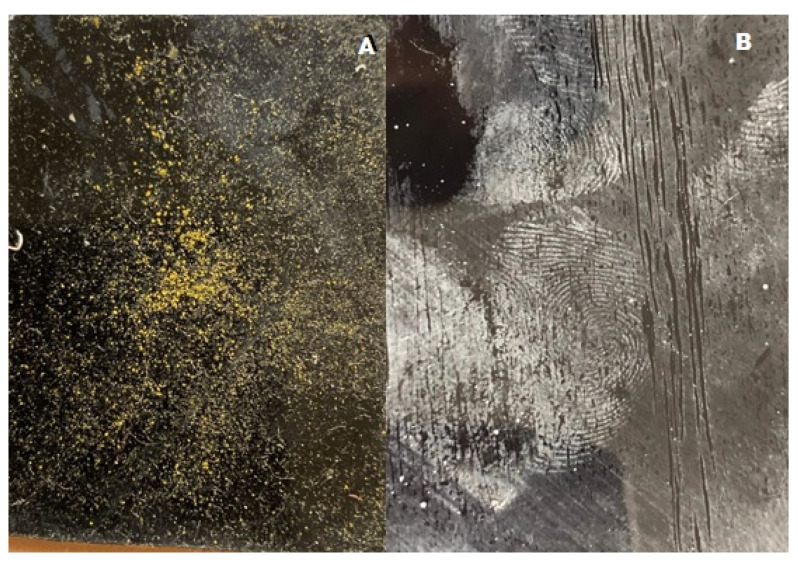
Comparison between the visualization of fingerprints using: (**A**) Cur-SLNs; (**B**) ZnO powder. Image of the latent fingerprint on a glossy wooden surface using ultraviolet illumination conditions.

Figure 5A,B display the fingerprint development outcomes utilizing Cur-SLNs in comparison to commercial ZnO powder. Compared figures can reveal that ZnO could effectively produce distinct ridges with a stronger contrast against the substrate background. Because the ZnO powder was more surface-adhesive, it performed well. Cur-SLN powder shows adhesion to fingerprints even with the smallest amount of moisture on the material. The creation of hydrogen bonds between the carbonyl and hydroxyl groups of the curcumin component Cur-SLN powder and the fatty acids/glycerides of sebum can be employed to assess the adherence of Cur-SLNs [18].

The slightest moisture on the Cur-SLN powder might smear the print and cause a loss of ridge definition, despite the fact that the Cur-SLNs have higher adhesion to the surfaces due to both hydrogen bond creation and their nanoscale size [55], which results in stronger adherence to the surface.

Since temperature and humidity are the causes of variance, further research should be done on how fingerprints form in different conditions and under controlled low-humidity conditions where Cur-SLN performance may be competitive with ZnO. Future work as comparative analyses on multi-surface and multi-evaluation conditions will be assessed.

## 3. Materials and Methods

### 3.1. Materials

Serchie (Youngsville, NC, USA) graciously supplied zinc oxide (ZnO). We bought Span 60, Stearic acid, and Pluronic F127 from Sigma Aldrich (St. Louis, MO, USA). For all other purposes, analytical-grade materials were utilized. There was no need for further purification because all of the chemicals and solvents utilized were of commercial reagent grade.

### 3.2. Preparation of ZnO and Cur-Loaded SLNs

A modified hot homogenization and high-speed stirring ultrasonication method was used to create SLNs [56]. After many exploratory investigations, the desired medication quantity (API; curcumin or ZnO) was combined with 100 mg of stearic acid as a solid lipid. The combination was then heated and kept at 80 °C to create a clear, uniform oil phase. Concurrently, 10 milliliters of distilled water with the required surfactant amount; SAA (either Pluronic F127 or Span 60) were heated to 85 °C, producing the aqueous phase. Using a magnetic stirrer, the aqueous phase was introduced to the lipid phase dropwise and stirred for ten minutes. In finality, a probe sonicator (GE130, probe CV18, amplitude 40%, 200 W, 20 kHz; Newtown, CT, USA) was used to ultrasonically treat the pre-emulsion for five minutes while being magnetically stirred at 20,000 rpm. The formulations were then allowed to settle to room temperature. The same technique, without Cur and ZnO, was used to create blank SLNs. The dispersion was held for further investigation and kept at 4 °C throughout.

### 3.3. Experimental Design and Optimization of ZnO and Cur-Loaded SLNs

The Design Expert^®^ program (Stat-Ease, Minneapolis, MN, USA) version 13 was used to carry out the optimization formulation process, adopting Cur-SLNs and ZnO-SLNs using a full 2^3^ factorial design with duplicate runs (16 total experimental runs). The study concentrated on three factors (independent factors), as indicated in Table 5, which were represented as the Quality Target Product Profile (QTPP): X1: type of API (ZnO or Cur), X2: type of surfactant (SAA; Span 60 or Pluronic F127), and X3: amount of API (ZnO or Cur), all at two levels. In contrast, the responses (dependent factors) were the particle size (nm) (Y1), polydispersity index (Y2), and zeta potential (mV) (Y3), which were considered the critical quality attributes (CQAs). To assess the data from the replies and the variable’s effect on the development of Cur-SLNs and ZnO-SLNs, a statistical investigation was conducted using analysis of variance (ANOVA) at the 95% level of significance (*p* < 0.05). The best fitting model was developed using surface response and polynomial equations, with the anticipated residual and adjusted errors and predicted R^2^ values serving as the basis. Based on the greatest zeta potential (ZP), the lowest particle size (PS), and polydispersity index (PDI) value, the optimized formula was chosen.

### 3.4. Characterization of Cur-SLNs and ZnO-SLNs

#### Particle Size (PS), Polydispersity Index (PDI) and Z-Potential Analysis (ZP)

A Malvern Zetasizer 2000 (Malvern Instruments Ltd., Grovewood, UK) was used to quantify PS, PDI, and ZP for the developed nanoparticles. The measurements were conducted at 25 ± 1 °C following sufficient dilution (10 times with deionized water) [57]. The electrophoretic mobility of vesicles in an electrical field was used to calculate the ZP. Results were shown as mean ± S.D. after measurements were made in triplicate. Malvern Software Version 3.0 was used to determine the mean particle size, ZP value, and polydispersity index (PDI), which were used to show the results.

### 3.5. Optimization of Cur-SLNs and ZnO-SLNs

After all responses were analyzed, the correlation between the dependent (responses) and independent variables (factors) was articulated using Design-Expert^®^ software Version 7, (Design-Expert® 7, State-Ease Inc., Minneapolis, MN, USA), which produced optimized formulations based on the necessary constraints (goals).

### 3.6. Cytotoxicity Study

Eight-well cell culture plates were used to cultivate the human fibroblast-derived cancer cell line (hFB cells; SPL, Life Sciences, Seoul, Republic of Korea). The 10% fetal bovine serum, 100 units/mL penicillin, and 100 μg/mL streptomycin were all present in the medium. They were all kept at 37 °C in humidified air with 5% CO_2_. The chosen formulations, ZnO-SLNs and Cur-SLNs, were tested for cytotoxicity. ZnO market powder was applied to the positive control cells. For an entire day, each was introduced individually into the cell culture medium [39].

#### Cell Viability Assay

A previously reported sulforhodamine B test technique (SRB) was used to measure cell viability in order to evaluate cellular proliferation [41]. To explain it simply, hFB cells were seeded at a density of 10^4^ cells/well on 8-well cell culture slides, and incubated at 37 °C with humidified 5% CO_2_. Untreated cells showed full growth (100%) and were used as a negative control. The viability of cancer cell lines was conducted at different concentrations gradient of the tested formulations (all containing an equivalent concentration) separately. To calculate IC50, or the inhibitory concentration (50%) [41]. The samples were adjusted for a final concentration at the IC50 concentration. Following the appropriate incubation period, the cells were stained with acridine orange (100 μg/mL) and ethidium bromide (100 μg/mL) dual stain dissolved in phosphate-buffered saline (PBS) at equal volume, both of which were obtained from Merck KGaA (Darmstadt, Germany), in order to examine the mode of cell death. A fluorescent microscope (AxioImager Z2, Zeiss, Jena, Germany) was used to stain and examine the cells. The green hue of living cells was used to identify them. In contrast, cells with yellow, orange, or red colors were categorized as necrotic, late apoptotic, or early apoptotic, respectively.

### 3.7. Evaluation of Optimized formulations

#### 3.7.1. Morphology Characterization of the selected formulations

A transmission electron microscope (TEM) (Joel JEM 1230, Tokyo, Japan) was used to determine the ultimate shape of solid lipid nanoparticles. A thin layer of one drop of the optimum nano-carrier (without dilution) was applied to a carbon-laminated copper grid and colored with 1.5% phosphotungstic acid [57].

#### 3.7.2. Raman Confocal Spectroscopy

A confocal Raman microscope system with a 532 nm frequency-doubled Nd: YAG laser as the excitation source was used to conduct Raman spectroscopy experiments. A thermoelectrically cooled CCD detector operating at −70 °C and an 1800 lines/mm holographic grating with 2 cm^−1^ spectral resolution was integrated into the system. The laser beam was focused onto the sample surface using a 50× long-working distance objective (numerical aperture = 0.50), producing an estimated spot size of 2 μm in diameter.

To balance sample integrity and signal quality, spectral collection settings were tuned. To avoid thermal deterioration or photochemical alteration of heat-sensitive components, the laser intensity at the sample was kept at 5 mW. Three accumulations were averaged to improve the signal-to-noise ratio after each spectrum was obtained using a 30 s integration time. This resulted in a total collection time of 90 s per measurement. The 400–3200 cm^−1^ spectral range, which includes fingerprint area and functional group characteristic frequencies, was recorded at sample intervals of 2 cm^−1^.

Prior to analysis, all samples were equilibrated for at least two hours under standard laboratory conditions (23 ± 2 °C, 45 ± 5% relative humidity) [48]. To evaluate spectral reproducibility and geographic homogeneity, measurements were made in triplicate on several sample sites. Using methods provided by the vendor, cosmic ray artifacts were automatically detected and eliminated during data collection. Spectral differences among crystalline Cur, stearic acid, Span 60, the physical mixture and the SLN formulation were systematically evaluated to identify molecular-level interactions and structural modifications associated with nanoparticle formation.

### 3.8. Effect of Storage Conditions on Optimized formulation

For three months, the optimized Cur-SLNs were kept at 25 °C and 4 °C, respectively. The PS, PDI, and ZP were used to examine the removed samples. To detect any signs of particle aggregation or sedimentation, a visual check was also carried out. The results obtained were statistically assessed using *t*-test analysis. The trials were repeated three times to guarantee accuracy, and the results were shown as mean ± SD [58].

### 3.9. Clinical Study of Curcumin SLNs for Fingerprint Detection in Forensic Medicine

To evaluate the efficiency of curcumin nanoparticles for fingerprint detection in forensic medicine, the following clinical study was carried out in the Department of Forensic Medicine, Faculty of Medicine, Misr University for Science and Technology, 6th of October City. As a rule of thumb, 12 participants per group (24 volunteers in both groups aged between 20 and 39 years old) participated to evaluate the efficacy and the clarity of fingerprint detection of the prepared Cur-SLNs compared to the marketed ZnO powder.

All participants were clearly informed about the study methods and aspects. Participants were informed that their participation was completely voluntary. Additionally, all ethical issues were considered, including the confidentiality and privacy of participants’ data [59]. Their data were collected from the Forensic Medicine and Clinical Toxicology Department, Faculty of Medicine, Misr University for Science and Technology, for the purpose of the study, which was approved by the university ethics committee (institutional review board at Misr University for Science and Technology; MUST-IRB, 12 July 2023) and was done in accordance with the protocols authorized by the U.S. Department of Health and Human Services and operated under wide assurance No. FWA000255577 under approval number 2022/0080. All participants provided written informed consent prior to enrollment, understanding that their data confidentiality and privacy were maintained throughout the study in accordance with institutional ethical requirements.

#### Fingerprint Collection and Development Process

The prepared Cur-SLN dispersion was pre-frozen at −22 °C for 24 h, then transferred to a freeze-dryer to obtain a dry powdered form (freeze dryer; LABCONCO, Singapore). The lyophilization cycle included an initial freezing phase at −30 °C for 4 h, followed by a primary drying stage lasting 20 h under a vacuum pressure of 50 mTorr, with the condenser temperature maintained at −50 °C. The resulting lyophilized material was preserved in tightly closed containers and kept at ambient temperature in desiccators containing anhydrous calcium chloride, which provides a relative humidity of approximately 29%, until further use.

Prior to fingermark collection, participants were instructed to wash their hands thoroughly using soap and dry them with paper towels. To ensure the transfer of well-defined sebaceous residues, participants gently rubbed their fingertips over naturally oily areas of the skin, including the regions behind the ears, the forehead, and the nose. For fingerprint development, small quantities of both commercially available and test powders were utilized. The powder was lightly applied onto the surface bearing the latent prints and then gently removed by tapping to eliminate excess material, thereby enhancing the visibility of ridge details. The latent prints are revealed by using powder brushes in soft, circular strokes. Fingerprint formation was conducted under standard laboratory conditions (temperature (±1): 25 ± 2 °C and relative humidity (±1): 55–60%) on a clean glossy timber surface (Sunmica) for comparison evaluation. Strong contrast against the dark backdrop, minor background discoloration, and excellent adhesion to fingerprint residues should all be present in the disclosed fingerprints. Prints were obtained from different finger patterns, including thumb, index, composite, arch, loop, and whorl types.

The developed fingermarks were documented using either a modified Nikon D6 camera equipped with a 20.8-megapixel sensor and the DCS5 imaging system (Foster & Freeman, Evesham, UK), or a Nikon D5500 digital camera (Nikon, Tokyo, Japan). The prints that showed consistent conformance and retained their quality over time would be eligible for in-depth analysis. The obtained photos were then examined using accepted methods for forensic analysis. Imaging was performed under both visible and ultraviolet illumination conditions. Ridge continuity, minutiae point clarity, background interference, and overall print definition were used to assess the quality of generated prints.

### 3.10. Statistical Analysis

The mean ± standard deviation (SD) of three replicates was used to express all results. Formulation design and evaluation were done using Design-Expert 7^®^ Software, version 7, Stat-Ease, Inc., Minneapolis, MN, USA. The effects of formulation parameters on the characteristics of the chosen formulations were assessed using a one-way ANOVA; *p* < 0.05 was deemed statistically significant.

## 4. Conclusions

The vast development in nanotechnology in all the sectors of the scientific fields, the traditional methods in latent fingerprint detection in forensic science could be replaced the current technologies. So, the developed nanoparticle system represents a “promising alternative” with potential forensic applicability pending further validation for latent fingerprint detection in forensic science. This study demonstrates, for the first time, that curcumin-loaded solid lipid nanoparticles (Cur-SLNs) can be formulated and optimized using a full 2^3^ factorial design to yield physicochemically stable nanoparticles (PS 221.55 nm, PDI 0.412, ZP +23.57 mV; desirability 0.971). Raman confocal spectroscopy confirmed successful curcumin encapsulation through characteristic peak shifts and broadening indicative of molecular dispersion and partial amorphization within the stearic acid/Span 60 matrix. TEM revealed spherical, non-aggregated nanoparticles consistent with DLS measurements. In cytotoxicity testing, Cur-SLNs produced a less necrotic cell death profile compared to ZnO-SLNs and commercial ZnO powder at equivalent concentrations, a finding with potential relevance to occupational safety—though the anti-proliferative activity of curcumin itself may confound direct safety comparisons in cell culture models. Lyophilized Cur-SLN powder demonstrated adhesion to sebaceous fingerprint residues. Cur-SLN nanopowder represents a promising, investigational nano-forensic reagent warranting further development, multi-surface validation, and quantitative performance benchmarking. Overall, the current findings provide an initial foundation supporting the potential applicability of Cur-SLN systems in safe latent fingerprint visualization, while also identifying opportunities for further optimization and broader evaluation in future studies.

## 5. Limitations and Future Work

Further studies are warranted to evaluate the long-term physical and chemical stability of the developed Cur-SLN system under various storage conditions, as well as to investigate its scalability and manufacturing feasibility for potential practical and industrial applications. As the clinical evaluation was conducted on a single surface type (glossy sunmica) under indoor ambient conditions, real crime scene substrates and environmental variables would be substantially more diverse. Moreover, broader evaluation on different substrate types and environmental conditions may further support the applicability of the developed system in forensic investigations. As the moisture sensitivity of Cur-SLN powder represents the most operationally significant limitation, surface humidity causes ridge smearing and loss of definition; future studies should systematically evaluate fingerprint development performance across defined humidity levels to establish operational boundaries and explore reformulation strategies to improve moisture resistance. In addition, employing fluorescence intensity quantitatively measurements and standardized forensic ridge clarity scoring (e.g., ENFSI or SWGMAT quality scales) would provide a more rigorous basis for comparison. Field validation with forensic practitioners is necessary to substantiate broader forensic applicability and support any claim regarding the replacement of conventional powders. The current study is a preliminary examination; further research is warranted.

## Figures and Tables

**Table 1:** Experimental runs, the composition and the measured responses of a 3Fl Factorial design for the preparation of different ZnO and Cur-loaded SLN formulations.

Run	Factors			Responses		
	A:X1: API	B:X2: Type of SAA	C:X3: Drug Amount	Y1: PS	Y2: PDI	Y3: ZP
1	ZnO	Span 60	100.0	371.3 ± 2.11	0.643 ± 0.02	17.9 ± 0.69
2	Cur	Pluronic F127	50.0	295.6 ± 1.98	0.649 ± 0.11	12.9 ± 0.65
3	Cur	Span 60	50.0	214.7 ± 2.01	0.416 ± 0.12	24.2 ± 1.03
4	ZnO	Pluronic F127	50.0	384.6 ± 2.67	0.735 ± 0.03	19.8 ± 1.11
5	Cur	Span 60	100.0	354.2 ± 2.34	0.430 ± 0.21	18.2 ± 1.04
6	ZnO	Span 60	50.0	320.8 ± 2.78	0.623 ± 0.07	21.2 ± 0.94
7	ZnO	Pluronic F127	100.0	430.1 ± 2.22	0.815 ± 0.07	16.8 ± 0.81
8	ZnO	Pluronic F127	50.0	384.6 ± 2.56	0.735 ± 0.09	19.8 ± 0.96
9	Cur	Pluronic F127	50.0	295.6 ± 1.98	0.649 ± 0.05	12.9 ± 0.78
10	Cur	Pluronic F127	100.0	375.3 ± 1.56	0.755 ± 0.06	12.3 ± 0.98
11	ZnO	Span 60	100.0	371.3 ± 2.12	0.643 ± 0.09	17.9 ± 1.09
12	Cur	Span 60	50.0	214.7 ± 1.32	0.416 ± 0.04	24.2 ± 1.09
13	Cur	Span 60	100.0	354.2 ± 2.19	0.430 ± 0.01	18.2 ± 1.18
14	ZnO	Span 60	50.0	320.8 ± 1.14	0.623 ± 0.03	21.3 ± 1.26
15	Cur	Pluronic F127	100.0	375.3 ± 1.18	0.755 ± 0.06	12.3 ± 1.11
16	ZnO	Pluronic F127	100.0	430.1 ± 1.29	0.815 ± 0.17	16.8 ± 1.23

PS: particle size, ZP: zeta potential, and PDI: polydispersity index.

**Table 2:** ANOVA summary and statistics results of all response variables.

Source	PS (nm)		ZP		PDI	
	F	*p*-Value	F	*p*-Value	F	*p*-Value
Model	120.32	<0.0001	51.25	<0.0001	1723.10	<0.0001
A-X1: API	213.65		23.42	0.0009	2815.63	
B-X2: Type of SAA	151.18		137.55	<0.0001	6231.13	
C-X3: Drug Amount	297.75		59.14		425.39	
AB	1.27	0.2886	75.68		659.85	
AC	45.49	<0.0001	0.022	0.8853	3.52	0.0935
BC	12.58	0.0060	11.66	0.0077	203.06	<0.0001
Std. Dev.	9.13		0.8420		0.0053	
Mean	343.32		17.92		0.6333	
C.V. %	2.66		4.70		0.8422	
R^2^	0.9877		0.9716		0.9991	
Adjusted R^2^	0.9795		0.9526		0.9986	
Predicted R^2^	0.9611		0.9101		0.9973	
Adeq Precision	33.3878		21.3672		115.3737	

**Table 3:** Polynomial Regression Equation for response to each of the tested factors.

	PDI	PS (nm)	ZP (mV)
**Intercept**	+0.633	+343.33	+17.92
A-X1: API	−0.071	−33.38	−1.02
B-X2: Type of SAA	−0.105	−28.08	+2.47
C-X3: Drug Amount	+0.028	+39.40	−1.62
AB	−0.034	+2.58	+1.83
AC	+0.003	+15.40	−0.031
BC	−0.019	+8.10	−0.719

PS: particle size, ZP: zeta potential, and PDI: polydispersity index.

**Table 4:** Effect of storage conditions on optimized Cur-SLNs.

	Fresh Prepared	After 3 Months, 4 C	After 3 Months, 25 C
PS	221.55 ± 1.446 nm	222.31 ± 1.915 nm	222.02 ± 1.682 nm
ZP	23.563 ± 0.947 mV	23.473 ± 1.262 mV	22.24 ± 1.950 mV
PDI	0.412	0.429	0.430

**Table 5:** Full experimental design parameters, levels, responses and desired constraints for product profiles for the preparation of Cur-SLNs and ZnO-SLNs.

QTPP: Factors (Independent Variables)	Low Levels	High Levels
**X1: Type of API**	ZnO	Curcumin
**X2: Surfactant type (SAA)**	Pluronic 127	Span
**X3: Amount of API**	50	100
CQAs: **Responses (dependent variables)**	**Desirability constraints**	
**Y1: PS (nm)**	Minimize	
**Y2: ZP (absolute values) (mV)**	Maximize	
**Y3: PDI**	Minimize	

PS: particle size, ZP: zeta potential, and PDI: poly dispersibility index.

## Data Availability

The original contributions presented in this study are included in the article. Further inquiries can be directed to the corresponding authors.

## Abbreviations

The following abbreviations are used in this manuscript:

PS	particle size
PDI	polydispersity index
ZP	zeta potential value
NPs	nanoparticles
SLNs	solid lipid nanoparticles
Zinc oxide	ZnO
Cur	curcumin
DLS	dynamic light scattering
PF127	Pluronic F127
API	medication
SAA	surfactant
QTPP	Quality Target Product Profile
CQAs	critical quality attributes
SRB	sulforhodamine B test technique
PBS	phosphate-buffered saline
TEM	transmission electron microscope
SD	standard deviation
IC50 values	the inhibitory concentration (50%)
